# Streamlined spectroscopic assay for besifloxacin: A one-pot approach evaluating drugs in eye drops and aqueous humor samples based on fluorescent isoindole generation. Comprehensive evaluation of whiteness, and blueness

**DOI:** 10.1038/s41598-026-41683-0

**Published:** 2026-04-21

**Authors:** Ahmed A. Abu-hassan

**Affiliations:** Department of Pharmaceutical Analytical Chemistry, Faculty of Pharmacy, Assiut Branch, Al-Azhar University, Assiut, 71524 Egypt

**Keywords:** Besifloxacin, Spectrofluorimetric, Eye drops, aqueous humor, Determination, Biological techniques, Chemistry, Drug discovery, Medical research

## Abstract

Besifloxacin (BSX) belongs to fluroquinolone-category antibiotic and marketed as ophthalmic suspension in the form of eye drops. BSX exert bactericidal by inhibiting DNA gyrase and topoisomerase IV which lead to retrogradation of cell division. The approach relies on the modification of BSX in an alkaline environment by tagging with o-phthalaldehyde. The fluorescent core was monitored using fluorimetric techniques at 434.6 nm after setting excitation at 337.9 nm. A proportional relationship was detected in the concentration range of 40–1100 ng mL^− 1^ for BSX, with values of detection and quantification limits equal to 12.8 and 38.7 ng mL^− 1^, respectively. The technique underwent validation and assessment following the standards outlined by the ICH. Furthermore, each experimental variable was meticulously examined and fine-tuned. The findings exhibit notable conformity to the prescribed reference procedure without any apparent inconsistencies between the two approaches. The designed probe shows good performance in the assay of BSX in eye drops and spiked aqueous humor. Finally, the whiteness and blueness of the methodology were assessed using corresponding metrics.

## Introduction

Fluoroquinolones are a class of synthetic antibacterial medications that are characterized by their core organic halogen structure and have garnered significant attention for their potent, broad-spectrum efficacy and versatile chemotherapeutic applications^1–3^. In this scenario, besifloxacin is particularly notable. It is a fluoroquinolone specifically approved for ophthalmic use and has been recently introduced to the Egyptian market. Besifloxacin ( 7-[(3*R*)-3-aminoazepan-1-yl]-8-chloro-1-cyclopropyl-6-fluoro-4-oxoquinoline-3-carboxylic acid) is highly effective against the main bacteria responsible for infectious conjunctivitis, including Staphylococcus aureus, Staphylococcus epidermidis, Streptococcus pneumoniae, and Haemophilus influenzae. Cases of resistance to this fluoroquinolone are uncommon, which makes it especially valuable in current clinical practice. Besifloxacin is available as its hydrochloride salt, marketed by Orchidia Pharmaceutical under the brand name Ocubesiflox™, in a 0.6% ophthalmic suspension^4^. Like other fluoroquinolones, besifloxacin works by binding to the bacterial enzymes DNA gyrase and topoisomerase IV, which play critical roles in bacterial DNA replication. By inhibiting these enzymes, besifloxacin disrupts the processes of transcription, replication, and the separation of bacterial chromosomal DNA during cell division^5^. Its dual-target approach reduces the development of resistance while increasing efficacy when compared to previous fluoroquinolones. The negative effects are usually restricted to localized discomfort, and it is often well tolerated^6^.

The literature review of BSX reveal variety of approaches for measuring the medication. Three precise and selective stability-indicating methods were developed and validated for quantifying besifloxacin hydrochloride in the presence of its peroxide degradants in both bulk and pharmaceutical formulations. The first method employed first-derivative (D1) spectrophotometry, measuring the peak amplitude at 258.6 nm, which demonstrated linearity from 2.5 to 80 µg/mL and a mean recovery of 100.6% ± 0.550. The second method utilized the first derivative of the ratio spectra (DD1) at 353 nm across the same concentration range, achieving a mean recovery of 99.91% ± 0.986. The third method was a TLC-densitometric technique that separated BSX on silica gel plates using a mobile phase of methanol: chloroform: ammonia: toluene: water (8:2:2:2:2 v: v:v: v:v), with quantitative analysis performed at 275 nm^7^. The measurement of BSX in the UV region using distilled water as solvent was reported and λmax was 289 nm^8^. Several fluorimetric methods for the analysis of BSX have been described in the literature. These techniques include derivatization reactions, such as condensation with the Hantzsch reagent and fluorescamine^9,10^, complexation with acetic acid^11^ or zinc^12^ and the enhancement of the drug’s intrinsic fluorescence through micellar enhancement using sodium dodecyl sulfate (SDS)^13^. Other methods involved UHPLC^14^, HPLC^15–17^, and electrochemical method^18^.

Spectrofluorimetry offers a significant analytical advantage due to its exceptional sensitivity and specificity in detecting fluorescent compounds, even at trace levels. This technique enables a linear response over a wide concentration range, making it ideal for both qualitative and quantitative analysis in fields such as pharmaceuticals, environmental monitoring, and biomedical research^19^. While spectrophotometric and chromatographic methods are widely used for pharmaceutical analysis, they are often associated with significant limitations. Spectrophotometric techniques generally suffer from lower sensitivity and selectivity, especially in complex matrices such as biological fluids, where interfering substances can compromise accuracy. Chromatographic approaches, though highly sensitive, typically require costly instrumentation, extensive sample preparation, time-consuming procedures, and the use of large volumes of organic solvents, which raise environmental and economic concerns. Furthermore, these methods may not always align with the principles of Green Analytical Chemistry due to their higher energy consumption, waste generation, and operational complexity^20,21^. These drawbacks highlight the need for alternative analytical strategies that are simpler, more eco-friendly, and cost-effective without sacrificing performance.

This study aims to introduce a straightforward and sensitive spectrofluorometric method for BSX analysis, employing tagging of BSX with o-phthalaldehyde with the aid of thiol. The high reactivity with PBX’s primary amino group resulted in a stable fluorescent derivative. Compared to previous methods, this new approach offers simplicity, and eliminates the need for complex instruments or harmful solvents, thus being more environmentally friendly. The strategy was properly validated following ICH guidelines and employed for analyzing BSX content in pharmaceutical eye drops, ensuring compliance with standards for content verification.

### Chemicals and materials

The materials for this study were sourced from commercial suppliers. BSX, with a certified purity of 99.50%, was procured from EVA Pharma for Pharmaceuticals (Haram, Gaza, Egypt). The commercial ophthalmic solution, Ocubesiflox^®^ (6.0 mg/mL), was obtained from a local pharmacy in Zifta City, Egypt. All other solvents and reagents, including ethyl alcohol, methyl alcohol, acetone, acetonitrile, acetic acid, sodium hydroxide, and hydrochloric acid, were supplied by El-Nasr Chemical Co. located in Cairo, Egypt.

A 0.01% w/v solution of o-phthalaldehyde was prepared by dissolving 10 mg of the compound (purchased from Thermo Fisher Scientific, Massachusetts, USA) in a small volume of HPLC-grade methanol (Sigma-Aldrich, USA). This solution was transferred to a 100 mL volumetric flask and brought to the mark with distilled water. Separately, a 0.01% v/v solution was made by diluting 2-mercaptoethanol (acquired from VWR International, Pennsylvania, USA) with water.

### Parent solutions

To prepare the besifloxacin (BSX) master solutions, 25 mg of authentic BSX powder was mixed with 80 mL of doubly-distilled water in a calibrated 250 mL container. After jerking the flask, additional solvent was added to reach the fill line, resulting in a master solution of 100 µg/mL. This solution was then attenuated using the previous solvent to generate various experimental solutions.

## Experimental procedures

Initially, BSX (1 mL), 2-mercaptoethanol (0.8 mL), o-phthalaldehyde ( 1.2 mL), and 1.5 mL of a borate buffer modifier were each added to individual 10-mL standardized flasks. These derivatized solutions were then given 15 min to stand at room temperature. A high-quality methanol was used to fill the remaining space in the flasks up to the indicated line. Subsequently, the isoindole derivative was excited at 337.9 nm and monitored fluorimetrically at 434.6 nm. To obtain the accurate emission value, the readings from the simultaneously prepared and monitored blank were subtracted from the sample readings acquired.

### Assay of BSX in eye drop sample

For the analysis of BSX in the eye drop formulation, a 3.33 mL aliquot of Ocubesiflox^®^ was placed into a 100 mL volumetric flask. Approximately 60 mL of distilled water was added, and the mixture was agitated to ensure complete mixing. The solution was subsequently diluted to the 100 mL mark with additional distilled water. This stock solution was filtered multiple times until clarity was achieved. Appropriate dilutions of this clear filtrate were then made to yield BSX concentrations within the analytical method’s quantifiable range. These diluted samples were analyzed according to the fluorescence protocol detailed in section “Experimental procedures”.

### Assay of BSX in spiked artificial aqueous humor

The synthesis of artificial aqueous humor was carried out in accordance with the procedure described by Fekry et al.^22^. Briefly, a test solution was formulated by dissolving requisite amounts of electrolytes, glucose, urea, albumin, and other constituents in water. The pH of the mixture was adjusted to 7.21 using 1.0 M HCl, followed by filtration and storage at -20 °C until required. To minimize potential matrix effects, a 100-fold dilution of the artificial aqueous humor with distilled water was prepared. Aliquots (1.0 mL) of this diluted solution were then transferred and mixed with known volumes of a Bfln.HCl standard to yield final concentrations of 2.5, 5, 7.5, and 10 µg mL⁻¹. The general assay procedure was conducted in this concentrations using 10-mL flask container.

## Results and discussion

**Fig. 1:**
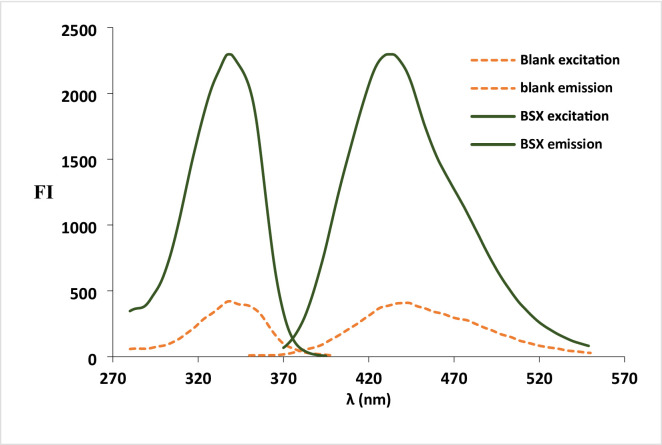
Excitation and emission spectra of BSX (500 ng/mL) and its reaction product with OPA.

**Scheme 1:**
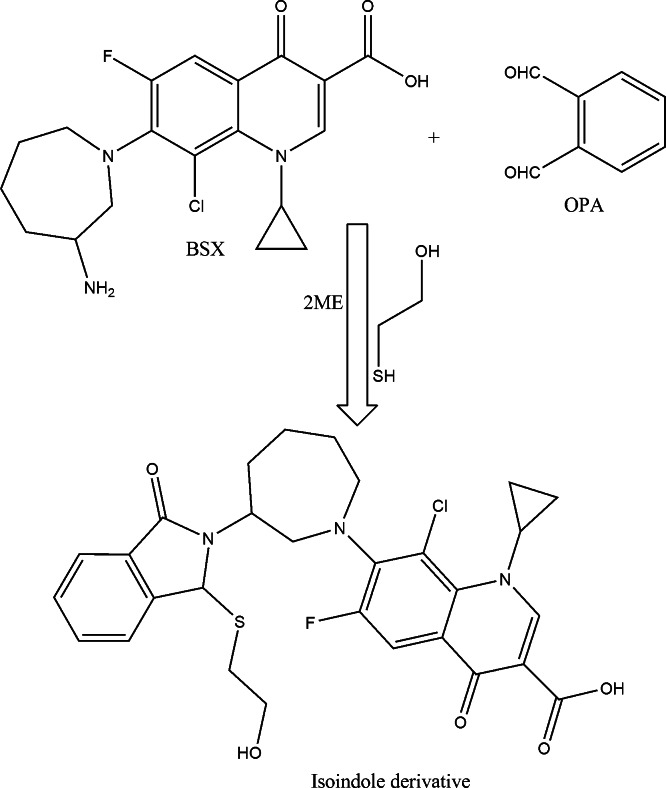
The pathway for isoindole derivative formation between BBX and OPA/2ME.

Several analytical methodologies rely on derivatizing a primary amino group using a fluorogenic reagent. Commonly employed reagents include Ninhydrin^23,24^, NBD-Cl^25,26^, NQS^27^, acetylacetone^28,29^, fluorescamine^30^, and o-phthalaldehyde^31,32^. Among these, o-phthalaldehyde stands out as the preferred choice due to its elimination of extraction steps seen in NQS or heating procedures required by NBD-Cl, NQS, Ninhydrin, and acetylacetone. Additionally, o-phthalaldehyde offers cost advantages over NBD-Cl and fluorescamine. The inclusion of thiol-containing compounds like 2-mercaptoethanol facilitates the condensation of the amino moiety of BSX with OPA, producing an isoindole fluorescent derivative. This reaction occurs under basic pH conditions, with the addition of 2-mercaptoethanol crucial for maintaining the stability of the isoindole product. Monitoring of the BSX derivative at specific excitation and emission wavelengths (λex 337.9 nm, λem 434.6 nm) (Fig. 1) and a schematic diagram (Scheme 1) elucidates the mechanism behind isoindole formation. Successful implementation of this methodology on BSX formulations and aqueous humor has been achieved following thorough validation procedures.

### Control of the used probe settings

A comprehensive analysis was conducted to scrutinize and control all factors influencing the stability and completion of the reaction. Keeping the existing conditions constant for all other elements, the RFI was gauged, and the influence of the specific factor was closely monitored. This study encompassed the examination of multiple variables including the OPA/2-ME reagent system, and buffer system which impact the pH of the derivative environment, dilution of reaction products with diverse polar and non-polar solvents, as well as the duration of the reaction.

**Fig. 2:**
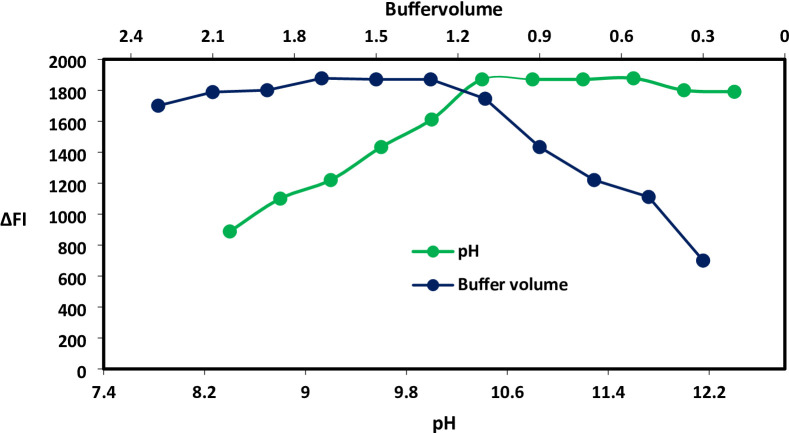
Impact of various pH and buffer volumes in the isoindole derivative formed from interaction of BSX (500 ng/mL) and OPA.

The examination of the fluorescence intensity of the isoindole derivative across a range of pH solutions (pH 8.4–12.4) was conducted using borate buffer. The formation of isoindole was observed to progress at alkaline pH levels, with the highest intensity achieved at pH 10.8 (as illustrated in Fig. 2). Additionally, the impact of borate buffer volume was investigated through a series of experiments involving varying volumes (from 0.3 mL to 2.3 mL). Analysis of the data, as depicted in Fig. 2, indicated that the optimal volume of borate buffer is 1.5 mL.

**Fig. 3:**
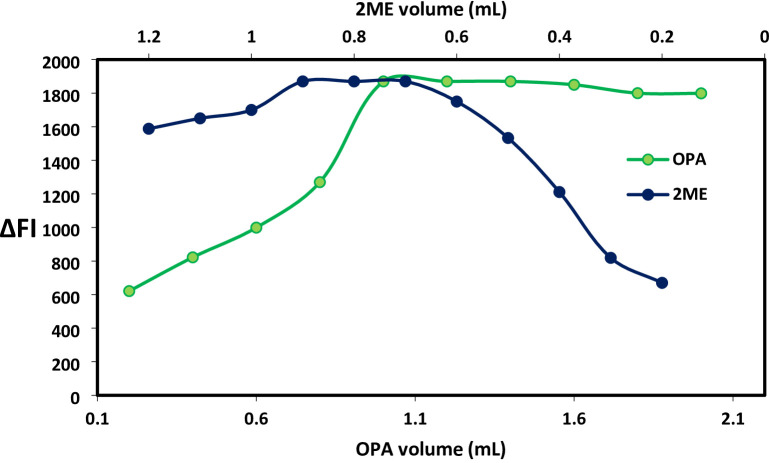
Impact of OPA and 2ME volume in the isoindole derivative formed from interaction with BSX (500 ng/mL).

The fluorescence response of the derivative was assessed by varying the volume of OPA across a 0.2–2.0 mL range. Fluorescence intensity progressively rose with increasing reagent volume, stabilizing within the 1–1.4 mL interval before exhibiting a modest decline at higher volumes, as illustrated in Fig. 3. To ensure consistent isoindole complex formation, the inclusion of 2-ME proved critical, with its volumetric optimization directly influencing signal stability. Experimental data revealed maximum fluorescence intensity when 0.8 ± 0.1 mL of 2-mercaptoethanol was introduced, as demonstrated in Fig. 3. This stabilization step was essential for maintaining reproducible fluorescence measurements throughout the analysis.

**Fig. 4:**
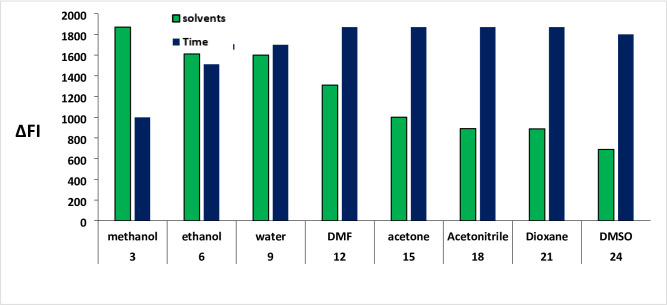
Impact of diluting solvent and time in the isoindole derivative formed from the interaction of BSX (500 ng/mL) and OPA.

The isoindole derivative resulting from the reaction of BSX with o-phthalaldehyde was subjected to dilution with various solvents in order to identify the most suitable one. Among the solvents examined, including dioxane, acetonitrile, water, DMSO, methanol, acetone, dimethylformamide, and ethanol (as Fig. 4), methanol demonstrated a notably high fluorescence value. Different time intervals (ranging from 3 to 24 min) were allocated for the formation of the isoindole derivative. The data presented in Fig. 4 indicated that 15 min proved to be the optimal duration for the formation of the product (isoindole derivative) with the highest fluorescence intensity.

### Validation of the system

In accordance with ICH guidelines, the probe was tested for key performance metrics such as linearity, precision, detection sensitivity, accuracy, selectivity, and overall adaptability.

### The linearity and range

To quantify BSX, a standard curve was established by graphing analyte concentrations versus the mean RFI response from triplicate samples. The data, analyzed by a least-squares regression model, confirmed a linear relationship within the 40–1100 ng/mL interval. Sensitivity, assessed per ICH criteria, defined the LOD and LOQ using the formulae 3.3σ/S and 10σ/S (where S = calibration slope and σ = standard deviation of the y-intercept). Based on this methodology, the LOD was determined to be 12.8 ng/mL and the LOQ 38.7 ng/mL. A full summary of the statistical analysis is provided in Table 1.

**Table 1:** Quantitative information used to support and validate the system’s creation.

Parameters	Values
Linear range (ng/mL)	40–1100
Slope	2.71
SD of the Slope	12.5
Intercept	463.28
SD of intercept (S_a_)	10.5
Correlation coefficient (r)	0.9995
Determination coefficients (r^2^)	0.9991
Number of determinations	7
Limit of quantitation (ng/mL)	38.7
Limit of detection (ng/mL)	12.8

### Accuracy and precision

This methodology was validated by analyzing various BSX concentrations within the established calibration range. Triplicate measurements for each concentration confirmed accuracy, which was quantified through recovery rates and standard deviation; the close agreement between found and added amounts is shown in Table 2. Method precision, assessed by analyzing three replicates at different levels, was found to be high, as indicated by low RSD values in Table 3, demonstrating the procedure’s reliability.

**Table 2:** The developed strategy’s accuracy at four different BSX concentrations.

Conc. level (ng/mL)	Recovery^*^± SD	% Error
100	100.39 ± 1.86	0.39
300	98.20 ± 0.94	1.80
500	98.72 ± 0.85	1.28
800	101.06 ± 1.87	1.06

*Mean of three BSX determinations and SD is the standard deviation.

**Table 3:** The two-level precision of the developed methodology.

Conc. level ( ng/mL )	Intraday precision		Interday precision	
	Recovery^*^± SD	RSD	Recovery^*^± SD	RSD
250	99.75 ± 1.70	1.71	98.96 ± 1.34	1.35
500	98.68 ± 0.92	0.94	98.56 ± 1.84	1.87
750	100.41 ± 0.99	0.98	101.64 ± 0.47	0.46

*Mean of three BSX determinations and RSD is the relative standard deviation.

### Robustness

A robustness study was conducted by intentionally varying critical parameters such as pH, buffer volume, OPA, and 2ME concentrations. As summarized in Table 4, the resulting percentage recoveries and their standard deviations were analyzed. The method proved to be robust, exhibiting consistent performance with low RSD values across all deliberate modifications.

**Table 4:** Evaluating the BSX assay’s present level of robustness.

Parameter	Value	% Recovery* ± SD
pH	10.6	98.36 ± 0.51
	11.0	98.61 ± 1.92
Buffer volume (mL)	1.4	101.90 ± 1.10
	1.6	99.22 ± 1.96
o-Phthalaldehyde volume (mL)	1.1	99.17 ± 1.72
	1.3	98.36 ± 1.51
2-mercaptoethanol volume (mL)	0.7	100.72 ± 1.22
	0.9	101.81 ± 1.11

*Mean of three BSX replicate measurements, SD = Standard deviation.

### Method blueness and whiteness

**Fig. 5:**
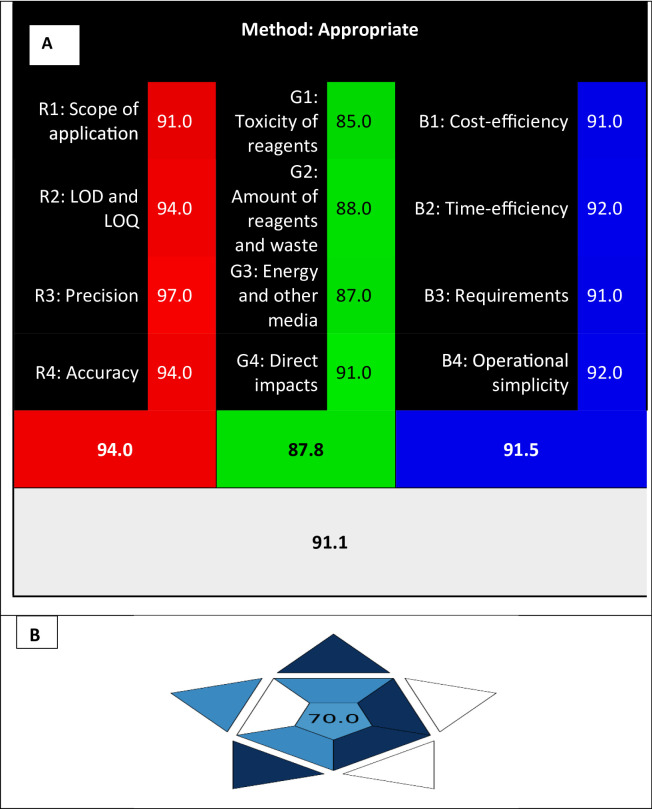
Evaluation of the fluorometric method using RGB12 (A), and BAGI (B) tools for Whiteness and blueness, showcasing the results.

Nowak and colleagues introduced the groundbreaking RGB12 algorithm in 2021, a trailblazing assessment tool for analyzing analytical strategies. This method evaluates methodologies through three distinctive criteria, each one denoted by a color-coded segment within the final assessment. The red zone meticulously examines the method’s credibility, encompassing precision, accuracy, LOD and LOQ, and the overall procedural suitability. The green part evaluates how well the procedure complies with the 12 Green Analytical Chemistry principles and confirms that it is environmentally friendly. In contrast, the blue region emphasizes the efficiency of the analytical methodologies, considering factors such as economic viability, practicality, time efficiency, and usability flexibility. Lastly, the white segment gauges the sustainability level based on the 12 principles of WAC, assessing the method’s overall balance and applicability for its designated analytical objective. The RGB12 tool offers a comprehensive and user-friendly assessment, facilitated by an accessible Excel spreadsheet that generates the evaluation results^33^. Figure 5A exemplifies the application of the RGB12 algorithm in evaluating the sustainability of a proposed approach across these four color-coded dimensions.

A novel metric tool, has been introduced to evaluate the viability of analytical techniques^34^. BAGI complements existing green metrics, concentrating primarily on the practical aspects of WAC. This innovative tool scrutinizes ten key attributes, comprising the essence of analysis, the ability to concurrently identify analytes, the throughput for sample analysis, the selection of reagents and materials, the requisite instrumentation, the efficiency in treating multiple samples concurrently, the necessity for preconcentration, the degree of automation, the methodology for sample preparation, and the sample volume. By appraising these attributes, a visual representation in the form of an asteroid pictogram is created, along with a corresponding score. The integration of BAGI promises to enhance the evaluation process of analytical methodologies, offering a comprehensive outlook on their practical utility and efficacy. Figure 5B clearly shows the practicability of the strategy.

### Application

#### Analysis of BSX in ophthalmic drops

The practical utility of the validated fluorimetric method was demonstrated by successfully quantifying BSX in a commercial eye drop formulation. As presented in Table 5, the recovery results obtained with the new method were compared against an established published procedure^9^. The data show no statistically significant differences between the two methods, confirming that the developed assay offers comparable accuracy and precision.

**Table 5:** Dosage form analysis of BSX and comparison with the reported method^9^.

Dosage form	Current method	Reported method	t- test value^b^	F-value^b^
	Recovery^a^ ± SD	Recovery^a^ ± SD		
Ocubesiflox^®^ (6.0 mg/mL) Eye drops.	98.91 ± 1.23	99.27 ± 1.43	0.34	1.36

^a^The value is the average of five determinations for both the proposed and reported methods.^b^ Tabulated values at 95% confidence limit are t = 2.306, F = 6.338.

### Monitoring of BSX in spiked artificial aqueous humor

The high sensitivity of the developed method was confirmed by quantifying BSX in artificial aqueous humor spiked with known concentrations of the analyte. The resulting emission responses yielded excellent recovery rates, ranging from 96.95 ± 2.93% to 98.42 ± 0.72% (Table 6). These near-quantitative recoveries demonstrate the method’s effectiveness for accurately determining BSX in this complex matrix.

**Table 6:** Determination of BSX in spiked artificial aqueous humor by the proposed method.

Conc. Level ( ng/mL )	Spiked aqueous humor Recovery*± SD
100	97.62 ± 4.12
300	96.95 ± 2.93
500	97.34 ± 2.29
800	98.42 ± 0.72

*Mean of three BSX replicate measurements, SD = Standard deviation.

## Conclusion

In this study, a novel, sensitive, and environmentally friendly fluorimetric method was developed for the quantification of besifloxacin (BSX) in ophthalmic formulations and simulated aqueous humor. The method is based on the derivatization of BSX with o-phthalaldehyde in the presence of 2-mercaptoethanol, resulting in a stable and highly fluorescent isoindole derivative. The proposed assay demonstrated excellent linearity over the range of 40–1100 ng mL⁻¹, with detection and quantification limits of 12.8 and 38.7 ng mL⁻¹, respectively. It was successfully validated following ICH guidelines and applied to commercial eye drops and spiked aqueous humor with high accuracy and precision. Furthermore, the methodology was evaluated using contemporary green, white, and blue assessment tools (RGB12 and BAGI), confirming its practicality, sustainability, and alignment with modern analytical eco-scale principles. Despite these advantages, certain limitations should be acknowledged. The method may be susceptible to interference from other primary amines in complex biological matrices, and its performance in undiluted real biological samples, such as human aqueous humor or tears, requires further validation. Overall, this one-pot fluorimetric strategy offers a reliable, cost-effective, and green alternative for the routine quality control and bioanalysis of besifloxacin in pharmaceutical and research settings.

## Data Availability

The data will be available upon reasonable request from the corresponding author.

## Abbreviations

BAGI: Blue Applicability Grade Index
BSX: Besifloxacin
ICH: International Council for Harmonisation
LOD: Limit of Detection
LOQ: Limit of Quantification
NBD-Cl: 4-Chloro-7-nitrobenzofurazan
OPA: o-Phthalaldehyde
RGB12: Red-Green-Blue-12 (assessment tool)
TLC: Thin-Layer Chromatography
UHPLC: Ultra-High Performance Liquid Chromatography
WAC: White Analytical Chemistry
2-ME: 2-Mercaptoethanol
